# Building a prediction model for iron deficiency anemia among infants in Shanghai, China

**DOI:** 10.1002/fsn3.1301

**Published:** 2019-12-05

**Authors:** Jiali Zhang, Weiming Tang

**Affiliations:** ^1^ Fenglin Community Health Service Center Xuhui District, Shanghai China; ^2^ Dermatology Hospital Southern Medical University Guangzhou China

**Keywords:** breastfeeding, complementary food, iron deficiency anemia, prediction model

## Abstract

Iron deficiency anemia (IDA) is a common micronutrient deficiency worldwide in infants. Iron deficiency anemia, during infancy, can have long‐lasting detrimental effects on the immune and neural systems; the damage is irreversible. This study aimed to build a prediction model to predict the potential risk of IDA among infants. To collect relevant information for model building, we recruited 528 infants from Fenglin Community Health Service Center in Shanghai, China, and collected the information of infants and their parents by using a structured questionnaire. We also got the blood routine examination results of the infants. Then, we used a multilayer perceptron model (MLP) of the neural network model in IBM SPSS Modeler 18.0 to construct the prediction model. Of the 528 included infants, 80 (15.2%) of them had lower hemoglobin values (<110 g/L) and were finally diagnosed with IDA. Based on the accuracy of different models, the model with the highest accuracy rate (97.3%) was chosen, and all the preselected 26 variables were included in the model. After the modeling, the results indicated that the number of months of exclusive breastfeeding was the most important predictive variable, followed by the mother having anemia during pregnancy, and then the number of months of feeding the infant with iron‐fortified rice flour. The model has good sensitivity (100%) and specificity (100%). By using this model, we can predict the potential risk of an infant having IDA and can take the initiative to prevent iron deficiency through the improvement of feeding methods.

## Graphical abstract

This study aimed to build a prediction model to predict the potential risk of IDA among infants in Shanghai, China. After the modeling, the results indicated that the number of months of exclusive breastfeeding was the most important predictive variable, followed by mother having anemia during pregnancy and the number of months of adding iron‐fortified rice flour.

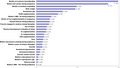

## 1 INTRODUCTION

The World Health Organization (WHO) reported that approximately 300 million children globally had anemia in 2011 and that iron deficiency is thought to be the most common cause of anemia (WHO, 2016a). This is true in developing countries, including China. The anemia prevalence rate of Chinese children under 5 years old in 2010 was as high as 12.6% (Ministry of Health & P.R. China, 2013). The Chinese “The National Nutrition Plan (2017–2030)” has advocated that effective intervention strategies should be implemented to bring down the anemia prevalence in children under 5 years old and pregnant women to 10% or lower by 2030 (General Office of the State Council & P.R. China, 2017). However, the necessary steps needed to reduce this anemia prevalence are still a significant public health challenge.

Iron deficiency anemia (IDA) is the most common form of anemia, especially among infants between 6–24 months, in China (Liu, Chen, & Zhao, 2011; Wang, Sun, & Chang, 2018). Iron is an essential nutrient for the development and cell growth of the immune and neural systems. It also helps in the regulation of energy metabolism and exercise (WHO, 2016a). Thus, IDA can have a significant impact on infant development (Baker, Greer, & Committee on Nutrition American Academy of P, 2010). For example, IDA can cause irreversible damage to an infant's long‐term neurodevelopment and psychomotor development, especially if IDA is severe and persistent (Beard, 2008; Peirano et al., 2009). Prevention and control of an infant's anemia can improve the nutritional status of childhood and the health of infants. Thus, identifying the factors associated with IDA among infants and providing relevant interventions is a research priority. Based on findings of different studies, the American Academy of Pediatrics (AAP) concluded that the risk factors for ID/IDA in infants were prematurity, low birth weight, exposure to lead, exclusive breastfeeding beyond 4 months of age without supplemental iron, and weaning to whole milk or complementary foods that do not include iron‐fortified cereals or foods naturally rich in iron (Baker et al., 2010). However, Chinese still have not an authoritative standard and guidance to prevent infant IDA. Thus, it is essential to develop a model that can predict IDA among Chinese infants. The results of the model can be used to guide the development of intervention strategies that can be used to prevent IDA in China. Furthermore, the plans for iron supplements for infants vary among different guidelines in China, and they do not often take the personal situation into consideration. Therefore, it is essential to evaluate the current condition of Chinese infants and provide individualized plans to improve their health.

This study aimed to build a predictive model of IDA among infants and to develop practical intervention strategies.

## 2 KEY MESSAGES

- Of the 528 included infants, 80 (15.2%) had lower hemoglobin values (<110 g/L) and were finally diagnosed as IDA.
- The final chosen predicting model has an accuracy rate of 97.3%, and all the preselected 26 variables were included in the model, with a sensitivity and specificity of 100%.
- The number of months of exclusive breastfeeding was the most important predictive variable, followed by mother having anemia during pregnancy, and then the number of months of adding iron‐fortified rice flour to the infant diet.

## 3 METHODS

A cross‐sectional study was conducted between October 2015 and August 2017 in Shanghai, China, to collect data that would be used in the prediction model.

### 3.1 Participants' recruitment

Infants who were taken to Fenglin Community Health Center (CHC, a primary care center) to get their 6‐month‐old examination were recruited (during 6–7 months). The inclusion criteria included participants currently living in Shanghai, willing to come to the CHC for IDA treatment in the coming one months if identified as an IDA case, and those between 6 and 7 months. Infants who were 8 months or elder were excluded. The parents who were willing to participate in this study signed a consent form and filled out one questionnaire. The study was designed with the plan to recruit 500 participants based on the assumption that the prevalence of IDA is 13% among infants in Fenglin Community Health Center, with an absolute error of 0.03, and a type one error of 5%.

### 3.2 Measures

A structural questionnaire was used to collect information from the parents of the infants (completed by one of the parents).

The information provided below was collected during the survey. For infants, we collected age (in days), gender (male vs. female), birth weight (2,500–3,000 g, 3,000–3,500 g, 3,500–4,000 g, or ≥4,000 g, based on birth certificate records), gestational age (<37 weeks, 37 weeks, 38–40 weeks, 41 weeks, or 42 weeks, based on birth certificate records), gravida (1, 2, 3 or 4 and above), para (1, 2, or 3), delivery mode (cesarean section, spontaneous delivery, or obstetric forceps), feeding patterns (exclusive breastfeeding, mixed feeding, artificial feeding), the age of complementary food (iron‐fortified rice flour) added (in months), and the supplementation of vitamin AD (VitAD, Yes or No), vitamin D (VitD, Yes or No), Ca (Yes or No), and Fe (Yes or No). For parents, we collected age (in years), educational level (high school or below, 2‐year college degree, bachelor's degree, master's degree or above), occupation, and annual income (in RMB). For the mothers, we further collected information on reproductive age (in years), anemia status during pregnancy (Yes or No), body mass index (BMI < 18.5 or >24), gestational hypertension (Yes or No), gestational diabetes (Yes or No), thyroid disease during pregnancy (Yes or No), passive smoking during pregnancy (Yes or No), and the supplementation of iron during pregnancy (Yes or No). The surveys were performed by self‐report of the infants' parents.

After the parents completed the questionnaires, the physicians at the CHC checked the questionnaire before the parents left the center. If there were any inconsistencies or incomplete information, the physicians would check further with the parents. In addition, the physicians conducted the physical development evaluation of the infants and collected the results of blood routine examinations. The physical assessment included weight, height, nutrition level, the physical development of the infant (moderate and severe malnutrition, mild malnutrition, normal weight, overweight, mild obesity, or moderate and severe obesity).

### 3.3 Laboratory test

After the parents finished the questionnaire, peripheral blood was collected from the left ring finger of the infant. The collected blood was sent to the laboratory for testing; the testing results were obtained in 10 min. The SYSMEX XS‐500i automatic blood cell analyzer was used to get hemoglobin values. During this procedure, medical services were provided to the IDA patients, and their respective IDA cases were verified.

In this study, we followed the WHO diagnostic criteria of anemia for a 6‐month‐old infant, which states as follows: An infant will be diagnosed with anemia if he/she has hemoglobin (Hb) < 110 g/L at the 6‐month visit. If infants had hemoglobin < 110 g/L, which were improved by iron supplementation after one month, they were considered to be IDA cases (Baker et al., 2010). The non‐IDA anemia cases (2 cases) were excluded from this study.

### 3.4 Statistical analysis

The data were double entered and verified by Epidata 3.0. After the data were cleaned, descriptive analysis was used to describe the distribution of the socio‐demographics of infants and their parents. Then, we generated different models using IBM SPSS Modeler 18.0, including neural network, Logistic regression, Bayes Net, and so on. Finally, the multilayer perceptron model (MLP) of the neural network model was selected, with the highest accuracy rate. Boosting algorithm was used to enhance the accuracy of the model further. The outcome for the model building was IDA among recruited participants, and all the other variables were treated as exposure variables. These variables include gestational age, birth weight, delivery mode, gravida, para, gender of infant, the number of months of exclusive breastfeeding, the number of months of complementary food (iron‐fortified rice flour) added, vitamin AD supplementation, vitamin D supplementation, Fe supplementation, Ca supplementation, physical development evaluation of infant, mother's reproductive age, mother's educational level, mother's years of living in Shanghai, whether mother had anemia, BMI < 18.5, BMI > 24, gestational diabetes, gestational hypertension, thyroid disease during pregnancy, weeks of iron supplementation during pregnancy, passive smoking during pregnancy of the mother, whether parents are engaged in medical‐related career, and parents' annual income.

A ROC curve and the gain curve were obtained by using IBM SPSS Modeler 18.0, and the sensitivity and specificity of the predictive model were obtained by using the same software.

## 4 RESULTS

A total of 528 infants were recruited and included in this study. Among these infants, 80 (15.2%) had lower hemoglobin values (<110 g/L) and were diagnosed as IDA. After using iron supplementary, their respective hemoglobin values increased.

### 4.1 Demographic characteristics

The 528 infants were 187 ± 7 days old (between 173–230 days, one was less than 6 months, and eight were recruited in 7th month). There were 271 (51.3%) male and 257 (48.7%) female infants. The mean birth weight was 3,398 ± 397 g. Overall, 514 of them (97.4%) were term infants; 239 infants (45.3%) had exclusive breastfeeding for six months, and 361 infants (68.4%) had added iron‐fortified rice flour. Only 13 (2.5%) of the infants were provided with Fe supplementation at the enrollment. Additionally, 329 (62.3%) of the infants had normal physical development, 139 (26.3%) were overweight, 38 (7.2%) were mildly obese, 2 (0.4%) were moderate and severe obesity, 13 (2.5%) had mild malnutrition, and 7 (1.3%) had moderate and severe malnutrition.

The childbearing age of the mothers was 30 ± 4 years old. A total of 396 (75.0%) of the mothers had one child. A total of 275 (52.1%) mothers had spontaneous delivery. In addition, 82 (15.5%) of them had anemia during pregnancy: 65 mild anemic cases, 9 moderately anemic cases, and 3 severe anemic cases. Five of the mothers did not remember the severity of their anemia during pregnancy. Overall, 416 (78.8%) mothers supplemented iron during pregnancy. A total of 74 (14.0%) of the parents engaged in a medical‐related career. Over 80% of the parents' education level (80.9% for mother, 81.2% for father) is above high school. For BMI, 20 (3.8) of mothers had a BMI > 24 during the pregnancy. 17 (3.2%) had gestational hypertension, and 45 (8.5%) had gestational diabetes (Table 1).

**Table 1:** Demographic characteristics of the infants and parents in Shanghai, China (*N* = 528)

Variables	Frequency	Percent (%)
Gender of the infants		
Male	271	51.3
Female	257	48.7
Birth weight (g) of the infants		
2,500–3,000	84	15.9
3,000–3,500	232	43.9
3,500–4,000	172	32.6
≥4,000	40	7.6
Gestational age of the infants		
<37 weeks	5	0.9
37 weeks	41	7.8
38, 39, 40 weeks	428	81.1
41 weeks	45	8.5
42 weeks	9	1.7
Gravida		
1	328	62.1
2	149	28.2
3	38	7.2
≥4	13	2.5
Para		
1	396	75.0
2	129	24.4
3	3	0.6
Delivery mode of the infants		
Cesarean section	232	43.9
Spontaneous delivery	275	52.1
Obstetric forceps	21	4.0
Feeding patterns of the infants		
Exclusive breastfeeding	239	45.3
Mixed feeding	266	50.4
Artificial feeding	23	4.4
Age of complementary food (iron‐ fortified rice flour) added of the infants		
4 months old or below	56	10.6
5 months old	186	35.2
6 months old	119	22.5
Never	167	31.6
VitAD supplementation of the infants		
Yes	296	56.1
No	232	43.9
VitD supplementation of the infants		
Yes	364	68.9
No	164	31.1
Ca supplementation of the infants		
Yes	118	22.3
No	410	77.7
Fe supplementation of the infants		
Yes	13	2.5
No	515	97.5
Physical development evaluation of the infants		
Moderate and severe malnutrition	7	1.3
Mild malnutrition	13	2.5
Normal	329	62.3
Overweight	139	26.3
Mild obesity	38	7.2
Moderate and severe obesity	2	0.4
Iron deficiency anemia of the infants		
Yes	80	15.2
No	448	84.8
Childbearing age of the mother		
<25	45	8.5
25–30	219	41.5
30–35	181	34.3
35–40	75	14.2
≥40	8	1.5
The educational level of the mother		
High school or below	101	19.1
2‐year college degree	105	19.9
Bachelor degree	248	47.0
Master degree or above	74	14.0
The educational level of the father		
High school or below	99	18.8
2‐year college degree	107	20.3
Bachelor degree	219	41.5
Master degree or above	103	19.5
Parents are engaged in medical‐related career		
Yes	74	14.0
No	454	86.0
Mother had anemia during pregnancy		
Yes	82	15.5
No	446	84.5
Mother supplemented iron during pregnancy		
Yes	416	78.8
No	112	21.2
Mother's BMI > 24 during pregnancy		
Yes	20	3.8
No	508	96.2
Mother's BMI < 18.5 during pregnancy		
Yes	13	2.5
No	515	97.5
Gestational hypertension		
Yes	17	3.2
No	511	96.8
Gestational diabetes		
Yes	45	8.5
No	483	91.5
Thyroid disease during pregnancy		
Yes	18	3.4
No	510	96.6
Mother had passive smoking during pregnancy^a^		
Yes	49	9.3
No	479	90.7
Parents' annual income (RMB)		
<50,000	38	7.2
50,000–100,000	102	19.3
100,000–200,000	154	29.2
200,000–500,000	186	35.2
≥500,000	48	9.0

a None of the mothers had active smoking during pregnancy.

### 4.2 Model selection and prediction model of iron deficiency anemia

After analyzing, the IBM SPSS Modeler presented ten different models, with an accuracy rate ranging between 76.5% and 97.3%. The model, which had the highest accuracy rate (97.3%), was finally chosen (Model 1, Table 2). Twenty‐six variables were selected for modeling based on previous studies. With the exception of the models we chose in this study, all the other models have accuracy rates of less than 87%, which are significantly lower than our current model.

**Table 2:** Accuracy rate of different models for predicting iron deficiency anemia among infants in Shanghai, China, 2015–2017 (*N* = 528)

Model	Accuracy rate	Predictive variable	Size	Record
1	97.3%	26	307	528
2	83.3%	26	422	528
3	80.5%	26	422	528
4	84.5%	26	542	528
5	86.6%	26	415	528
6	78.4%	26	297	528
7	76.5%	26	422	528
8	82.6%	26	356	528
9	81.3%	26	466	528
10	84.1%	26	415	528

**Figure 1:**
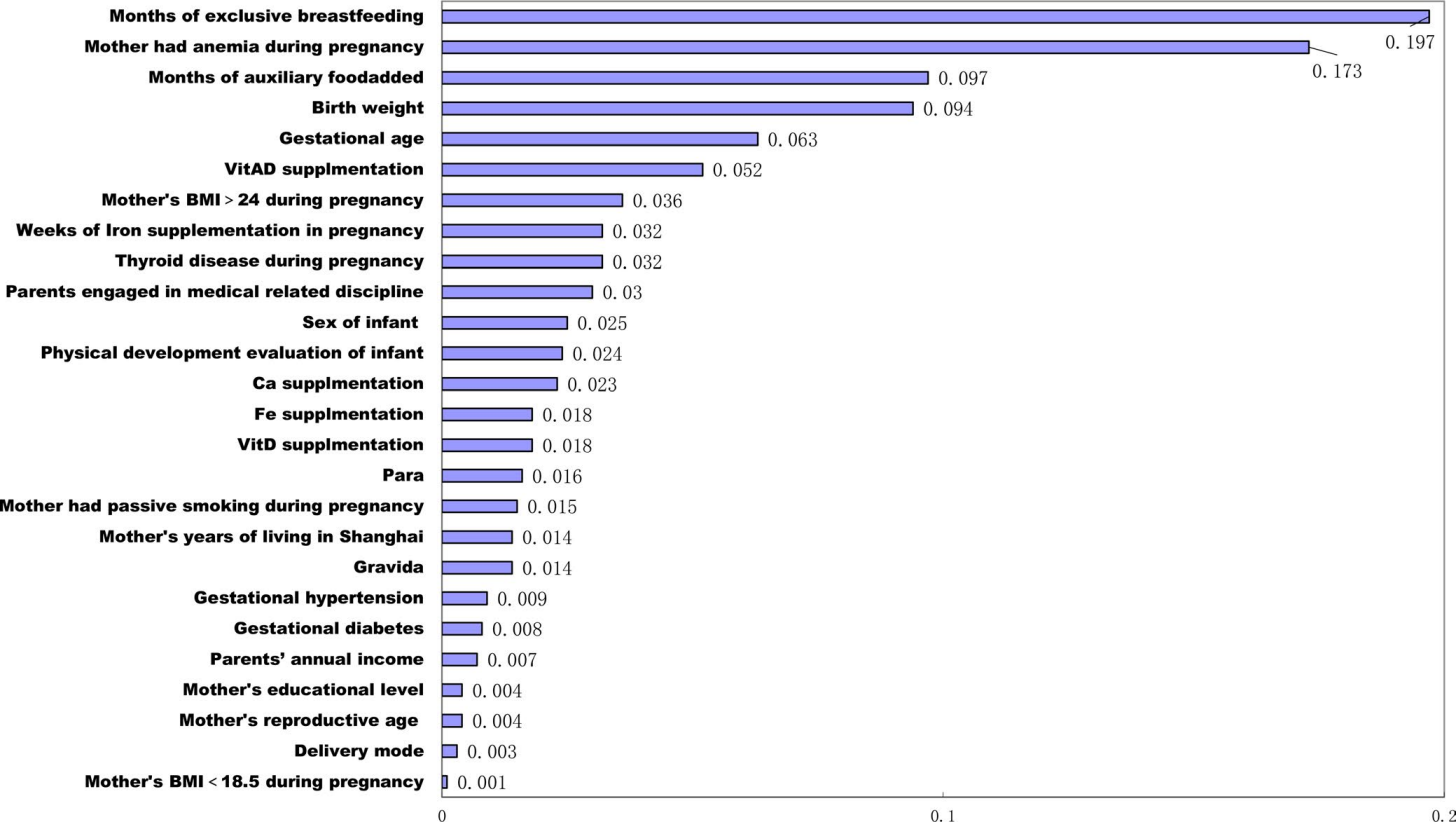
The ranking of different predictive variables in the final model in Shanghai, China, 2015–2017 (*N* = 528)

The relative importance of these variables is shown in Figure 1. We also calculated the weight of each included variable; the number of months of exclusive breastfeeding was the most important predictive variable, with a value of 0.197. Furthermore, mothers having anemia during pregnancy ranked second (0.173), while the third to fifth ranks was the number of months of adding iron‐fortified rice flour (0.097), birth weight (0.094), and gestational age (0.063), respectively. All the other factors had values of 0.05 or below.

### 4.3 Model verification

**Figure 2:**
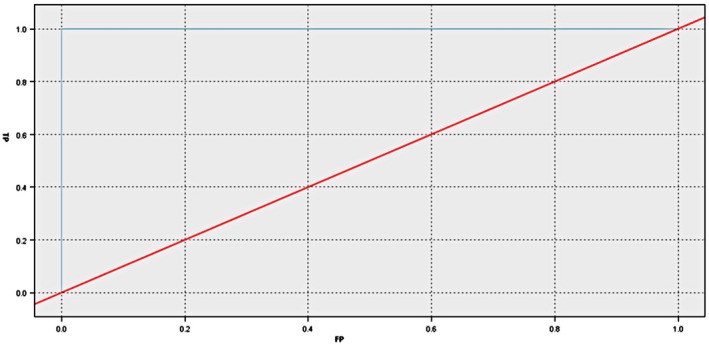
ROC curve of the selected prediction model for predicting iron deficiency anemia among infants in Shanghai, China, 2015–2017 (*N* = 528)

**Figure 3:**
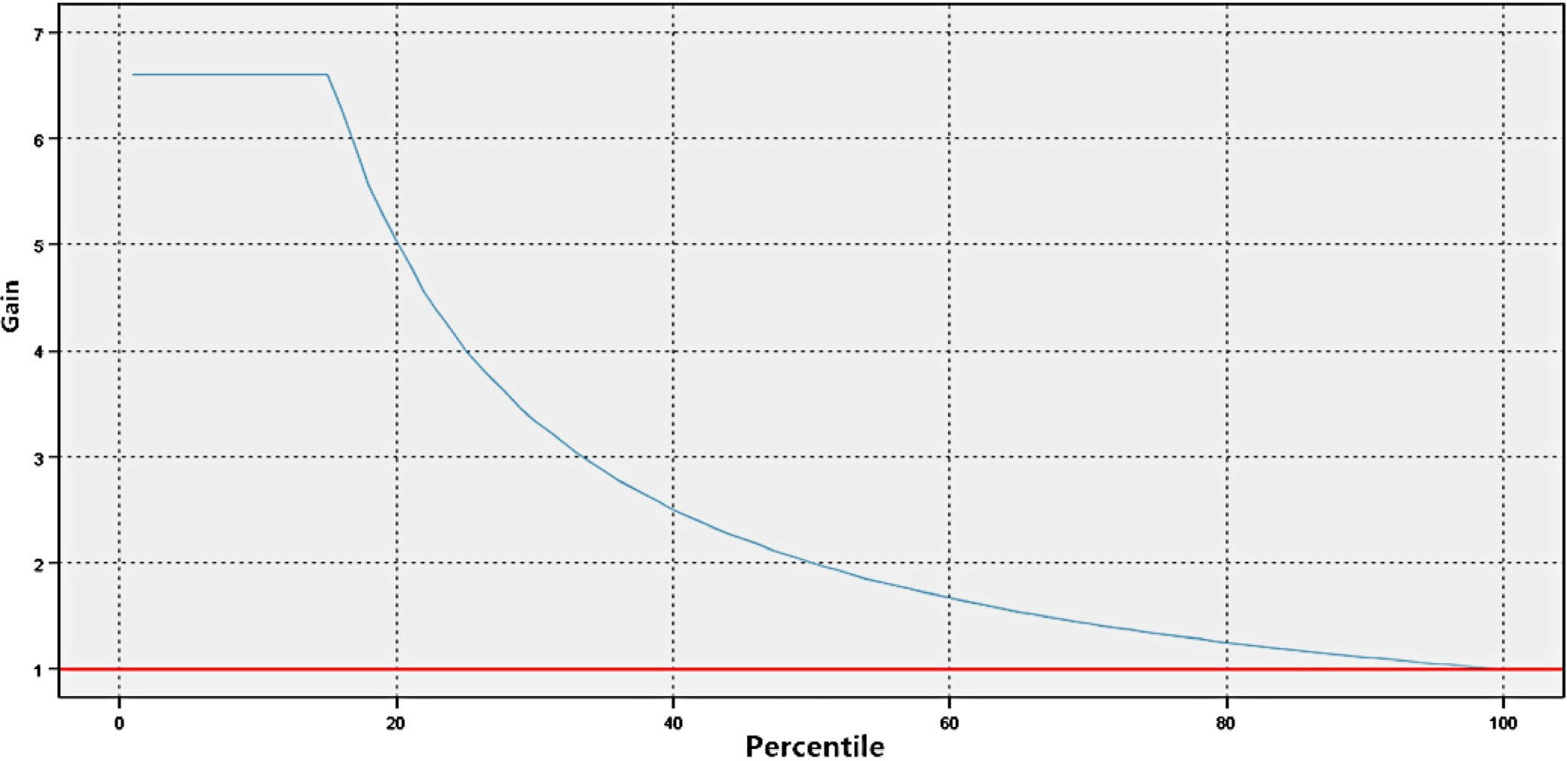
Gain curve of the selected prediction model for predicting iron deficiency anemia among infants in Shanghai, China, 2015–2017 (*N* = 528)

The model also gave us the results of the ROC curve (shown in Figure 2) and gained curve (shown in Figure 3). The results show that the finally selected model (Model 1 in Table 2) has extremely high sensitivity and specificity (100% for both), which indicate that the rates of misdiagnosis and missed diagnosis in this model can be neglected, based on this predictive model. The prediction effect is good, and the accuracy of classification is very high.

## 5 DISCUSSION

Knowing the variables that can predict the risk of having IDA accurately is essential for preventing IDA. We built a neural network model with high accuracy for predicting the risk of having IDA among infants at our center. The results of our study indicate that the number of months of exclusive breastfeeding, whether mothers had anemia during pregnancy, and the number of months of adding iron‐fortified rice flour rank in the top three standings. These findings can help develop practical intervention strategies.

The results of this study show that the number of months of exclusive breastfeeding is the most important predictive variable. The more extended the exclusive breastfeeding, the more likely the infant has IDA. This result is similar to a large number of previous studies (Burden et al., 2007; Capozzi, Russo, Bertocco, Ferrara, & Ferrara, 2010; Monterrosa et al., 2008). The normal infant can access sufficient iron from breast milk until the infant has doubled his or her birth weight, which occurs at about 4–6 months of age in a term, normal birth weight infant (Domellof, 2007). Although the iron absorption rate of breast milk is five times that of formula milk, the amount of iron in breast milk is still insufficient for infants (Li, 2011). But exclusive breastfeeding is recommended strongly for improving infant health because of the other nutritional components. From the data of the Chinese National Health Planning Commission, the rate of exclusive breastfeeding among the infants under 6 months old in rural areas and the urban area was as low as 30.3% and 15.8%, respectively (Center for Health Statistics & Information, 2009). This rate is far below the target rate suggested by the WHO in the 2025 Global Nutrition Targets, which is at least 50% (WHO, 2017). We should recommend exclusive breastfeeding according to WHO, but providing additional iron supply to exclusively breastfeeding infants proves essential and integral in preventing IDA.

We also found that maternal anemia during pregnancy is the second crucial predictive variable of IDA. The most recent estimates about anemia showed that the global prevalence of anemia, in 2011, among women of reproductive age was 29% (WHO, 2016b). Although the anemia rate during pregnancy in our study (15.5%) was much lower than this prevalence, the anemia rate among pregnant women in Shanghai is still high. WHO aimed to achieve a 50% reduction of anemia in women of reproductive age by 2025 (WHO, 2017); further reduction of anemia is a significant challenge. For infants, iron that is obtained from a mother during the fetal stage can generally meet their needs between 4 and 5 months of age. But if the mother has anemia during pregnancy, maternal iron storage decreases, transferrin receptor compensatory conversely increases, and the placental iron uptake capacity decreases. This reduces fetal iron gain among infants (Pasricha, Drakesmith, Black, Hipgrave, & Biggs, 2013). For such infants, the iron obtained from the mother is depleted more quickly, and the infant is more prone to anemia. Providing further iron supply to pregnant women is considered an essential prevention strategy.

The months of adding iron‐fortified rice flour ranked third among all the chosen variables. To meet the needs of the infants, adding complementary food timely and effectively is vital to prevent IDA (Domellof, 2007; Jonsdottir et al., 2012). The American Academy of Pediatrics (AAP) recommends exclusive breastfeeding infants should get a daily supplement of 1 mg/kg iron from 4 months old until the infant can intake adequate iron‐bearing foods to reduce iron deficiency (Baker et al., 2010). But some studies support that iron supplements are beneficial in iron‐deficient children, but there is a risk of incurring adverse effects in those who are iron sufficient (Domellof, 2010). In China, most of the parents are generally not accepting of iron supplements, so iron‐fortified rice flour can be used as a means of supplementing iron for infants of 4 months old and above (Jonsdottir et al., 2012; Qasem, Fenton, & Friel, 2015). Chinese Dietary Guidlines, (2016) point out that the first complementary foods for infants should be iron‐rich foods, such as iron‐fortified rice flour, minced meat, and so on.

Birth weight and gestational week were two other significant predictors. Previous studies have indicated that the last three months of pregnancy are a critical period of fetal intrauterine iron transport (Li, 2011). Iron is actively transported to the fetus through the placenta during pregnancy, and the fetus maintains a high intrauterine hemoglobin level. So, the fetus can adapt to the intrauterine environment, which relatively lacks oxygen. The larger the gestational week, the more iron the fetus gets. And there is about 75 mg iron per kilogram, so the more significant the body weight, the higher the iron content (Dewey & Chaparro, 2007). In other words, the larger the birth weight and gestational weeks, the higher the iron content, and the less likely the infant will have IDA.

The prediction model can be used to predict whether the infant will have IDA, based on the situation of the infants and their parents. This prediction model is a multilayer perceptron model (MLP) of the neural network model in the IBM SPSS Modeler 18.0. Boosting algorithm was used to enhance the accuracy of the model. The prediction effect is good, and the classification accuracy is very high. However, even though our model has sensitivity and specificity of 100%, over‐fitting is still a problem in our study, since the most stable condition of the neural network model is that the number of samples is 40–80 times the number of variables. We included 26 variables in our model, but we only recruited 528 participants. It is believed that this kind of model can be more stable through the increase of sample size.

In addition, our study has a few limitations. First, most CHCs do not have serum ferritin detection in Shanghai, and the physicians could only diagnose IDA by Hb < 110 g/L, and supplementary iron treatment is effective. Thus, we could not monitor the iron deficiency status of infants timely. Secondly, once physicians in CHCs found high‐risk infants (low birth weight, twins, premature birth), they had to refer the infants to a high‐level health center (Maternal and Child Health Center) for further checkup and treatment. The high‐risk infants were not be referred back to the CHCs until they had normal growth and development. That is the reason why only a few low birth weight infants, twins, and premature birth infants were included in this study. Thus, the results of this model need to be utilized with caution. Third, as stated earlier, over‐fitting is a problem in our study because of the lack of a large sample size. Our results, however, provide a preliminary result for the prediction of IDA, and we plan to continue to recruit more participants for further research.

## 6 CONCLUSION

The prediction model established in this study showed that exclusive breastfeeding, maternal anemia during pregnancy, and nontimely supplementation of complementary food were the top three risk factors for IDA in infants. WHO recommends exclusive breastfeeding before six months old, but iron supplementation should be added. Following strategies can be implemented to prevent IDA in infants: (a) We should enhance parents' acceptance of iron supplementation by health education; (b) we should continue to improve iron supplementation during pregnancy, even 78.8% participants had taken iron during pregnancy; (c) we should supervise and urge pregnant women with anemia to get treatment as soon as possible, to improve their iron nutrition; (d) for nonexclusive breastfeeding infants, we suggest they take complementary food timely and effectively to prevent IDA.

## CONFLICT OF INTEREST

None declared.

## ETHICAL APPROVAL

Ethical approval was obtained from the ethics review committees at the Fenglin Community Health Service Center (Shanghai, China) prior to the survey launch. Informed consent was obtained from all the participants by e‐signing the inform consent form online.
